# Amyloid Beta-Mediated Hypomethylation of Heme Oxygenase 1 Correlates with Cognitive Impairment in Alzheimer’s Disease

**DOI:** 10.1371/journal.pone.0153156

**Published:** 2016-04-08

**Authors:** Hye Youn Sung, Byung-Ok Choi, Jee Hyang Jeong, Kyoung Ae Kong, Jinha Hwang, Jung-Hyuck Ahn

**Affiliations:** 1 Department of Biochemistry, School of Medicine, Ewha Womans University, Seoul, Republic of Korea; 2 Department of Neurology, Samsung Medical Center, Sungkyunkwan University School of Medicine, Seoul, Republic of Korea; 3 Department of Neurology, Ewha Womans University Mokdong Hospital, Seoul, Republic of Korea; 4 Clinical Trial Center, Ewha Womans University Medical Center, Seoul, Republic of Korea; 5 Department of Biomedical Sciences, Seoul National University Graduate School, Seoul, Republic of Korea; 6 Department of Biochemistry, Tissue Injury Defense Research Center, School of Medicine, Ewha Womans University, Seoul, Republic of Korea; Queen's University Belfast, UNITED KINGDOM

## Abstract

To identify epigenetically regulated genes involved in the pathogenesis of Alzheimer’s disease (AD) we analyzed global mRNA expression and methylation profiles in amyloid precursor protein (APP)-Swedish mutant-expressing AD model cells, H4-sw and selected heme oxygenase-1 (HMOX1), which is associated with pathological features of AD such as neurofibrillary tangles and senile plaques. We examined the epigenetic regulatory mechanism of HMOX1 and its application as a diagnostic and prognostic biomarker for AD. Our results show that *HMOX1* mRNA and protein expression was approximately 12.2-fold and 7.9-fold increased in H4-sw cells, respectively. Increased *HMOX1* expression was also detected in the brain, particularly the hippocampus, of AD model transgenic mice. However, the methylation of specific CpG sites within its promoter, particularly at CpG located −374 was significantly decreased in H4-sw cells. Treatment of neuroglioma cells with the demethylating agent 5-aza-2′-deoxycytidine resulted in reduced methylation of *HMOX1* promoter accompanied by enhanced HMOX1 expression strongly supporting DNA methylation-dependent transcriptional regulation of *HMOX1*. Toxic Aβ-induced aberrant hypomethylation of *HMOX1* at −374 promoter CpG site was correlated with increased HMOX1expression. In addition to neuroglioma cells, we also found Aβ-induced epigenetic regulation of *HMOX1* in human T lymphocyte Jurkat cells. We evaluated DNA methylation status of *HMOX1* at −374 promoter CpG site in blood samples from AD patients, patients with mild cognitive impairment (MCI), and control individuals using quantitative methylation-specific polymerase chain reaction. We observed lower methylation of *HMOX1* at the −374 promoter CpG site in AD patients compared to MCI and control individuals, and a correlation between Mini-Mental State Examination score and demethylation level. Receiver operating characteristics analysis revealed good discrimination of AD patients from MCI patients and control individuals. Our findings suggest that the methylation status of *HMOX1* at a specific promoter CpG site is related to AD progression.

## Introduction

Alzheimer’s disease (AD) is a progressive neurodegenerative disorder that results in the death of nerve cells, deterioration of cognitive function [1], and eventual death from complications. It is the most common form of age-related dementia, with its incidence approaching 50% in people over 85 years of age [2].

With the rate of AD increasing rapidly due to the aging of the population, the need for early diagnosis, prevention, and treatment is growing. However, the diagnosis of AD relies mostly on neuropsychological tests such as the Mini-Mental State Examination (MMSE), which has low sensitivity among patients with early dementia [3], or specialized magnetic resonance imaging (MRI) and positron emission tomography scans, which are lengthy and costly [4]. Other potential diagnostic biochemical methods include detecting the level of amyloid beta (Aβ) or tau protein in the cerebrospinal fluid (CSF) [5–7] or serum [8, 9], or the level of glial fibrillary acidic protein [10]. However, the convenience and accuracy of these methods remain questionable.

Heme oxygenase 1 (HMOX1) is a heat shock protein 32 that exists in the endoplasmic reticulum. HMOX1 binds with NADPH cytochrome p450 reductase to convert the pro-oxidant heme to CO, Fe^2+^, and biliverdin, which is subsequently converted to the antioxidant bilirubin by biliverdin reductase [11]. *HMOX1* expression is induced by oxidative, nitrosative, and inflammatory stress, ischemia, heat shock, hemin, and Aβ [12, 13]. HMOX1 is highly expressed in hippocampal and cortical astrocytes and neurons in patients with AD or mild cognitive impairment (MCI), where it co-localizes with senile plaques, neurofibrillary tangles, corpora amylacea, and other factors [14, 15]. The degradation of heme to biliverdin and conversion of biliverdin to bilirubin protects cells from oxidative stress [16, 17]. In contrast to the protective effects of HMOX1, studies show that the up-regulation of HMOX1 increases oxidative stress through the accumulation of Fe^2+^ in mitochondria and other cellular compartments of astroglia and promotes mitochondrial damage and macroautophagy [18–20].

Evidence indicates that epigenetic deregulation is linked to AD progression and development [21, 22]. For example, the expression of AD-related genes such as amyloid precursor protein (APP), β-site APP-cleaving enzyme1, and Presenilin 1 is regulated by the epigenetic mechanism of DNA methylation in AD brains [23, 24]. Furthermore, AD brains exhibit high levels of the potential methyltransferase inhibitor S-adenosylhomocysteine, which is negatively correlated with cognitive function, suggesting the involvement of global hypomethylation in AD progression [25].

Herein, we investigated the APP-mediated alteration of HMOX1 expression and related epigenetic regulation mechanisms using glioblastoma cells harboring a Swedish mutation of APP. We detected a profound alteration in methylation status at the *HMOX1* promoter region and found that the expression of HMOX1 was regulated by DNA methylation status at this specific cytosine-phosphate-guanine (CpG) site in an AD model cell line. We also detected hypomethylation of *HMOX1* at this specific promoter CpG site in the blood of AD patients compared with that of controls. Cognitive impairment in AD patients was strongly correlated with *HMOX1* methylation status at this specific site. We therefore suggest that the methylation status of *HMOX1* at a specific promoter CpG site is related to AD progression.

## Materials and Methods

### Cell culture

Wild-type human glioblastoma H4 cells (H4) and APP695-Swedish mutant (K595N/M596L)-expressing H4 cells (H4-sw) were kindly provided by Sangmee Ahn Jo’s lab (Dankook University, Chungnam, Korea) and have been previously described [26, 27]. H4 and H4-sw cells were cultured in Dulbecco’s modified Eagle’s medium (Biowest, L0103-500) containing 10% fetal bovine serum (Gibco/Life technologies, 16000–044), 100 U/ml penicillin (Gibco/Life technologies, 15140–122) and 100 μg/ml streptomycin (Gibco/Life technologies, 15140–122) [28]. To maintain H4-sw cells, 500 μg/ml geneticin (Gibco/Life technologies, 10131–027) was added to the growth media.

A human T-cell leukemia cell line Jurkat, a human monocytic leukemia cell line U937, a human myelogenous leukemia cell line HL-60 and a human acute monocytic leukemia cell line THP-1 were purchased from the Korean Cell Line Bank (KCLB; Seoul, Korea). U937, HL-60 and THP-1 were cultured in suspension in RPMI 1640 medium containing 300 mg/l L-glutamine, 25 mM (4-(2-hydroxyethyl)-1-piperazineethanesulfonic acid (HEPES) and 25 mM NaHCO_3_ (Welgene, LM011-03), supplemented with 10% fetal bovine serum, 100 U/ml penicillin and 100 μg/ml streptomycin. Jurkat was cultured in suspension in RPMI 1640 medium containing 2.05 mM L-glutamine (Hyclone, SH30027.01) supplemented with 10% fetal bovine serum, 100 U/ml penicillin and 100 μg/ml streptomycin. The culture was kept in a humidified incubator with 95% air and 5% CO2 at 37°C and was subcultured at 1 × 10^5^ cells/ml every 3 to 4 days.

### Mice

APP swe/PS1 transgenic mice (B6C3-Tg(APP695)85Dbo Tg(PSEN1)85Dbo) were originally purchased from The Jackson Laboratory (www.jax.org) and subsequently bred in the animal care facility at the College of Medicine, Ewha Womans University. These mice doubly express human APP carrying Swedish familial AD-linked mutations (K670N/M671L) and human Presenilin 1 encoding a mutant exon 9-deleted variant (PSEN1/dE9). Twelve-month-old age-matched transgenic and wild-type littermates were used in the experiments. For euthanasia, mice were deeply anesthetized with isoflurane in a 20% O_2_/80% air mixture: 5% for induction, 2% for maintenance until insensate using a standard animal mask, then mice were sacrificed by cervical dislocation under anesthesia according to procedures approved by the Institutional Animal Care and Use Committee of Ewha Womans University School of Medicine (Permit Number: 12–0206). Brains were harvested, and the frontal cortex, hippocampus, and cerebellum were isolated and freshly used for gene expression analyses.

### Human blood samples

Human blood samples were collected from 105 patients with AD (age: 81.26 ± 5.44 years, MMSE score: 1~28), 13 patients with MCI (age: 73.62 ± 6.19 years, MMSE score: 16~26), and 10 control individuals (age: 72.10 ± 7.40 years, MMSE score: 24~29). Written informed consent was obtained from all participants and control individuals according to the protocol approved by the Institutional Review Board of Mokdong Hospital, Ewha Woman’s University and the Korea National Institute of Health (Permit Number: ECT12-14A-27) to publish these case details. For patients with impaired cognition, written permission was obtained from their family in accordance with the Declaration of Helsinki. All AD patients met NINCDS-ADRDA criteria [29]. Patients’ cognitive symptoms were evaluated using the Korean version of the MMSE [30], clinical dementia rating scale, and Seoul Neuropsychological Screening battery [31]. The diagnosis of AD was confirmed in all patients by the presence of temporo-parietal regional atrophy and the absence of significant vascular lesion in brain MRI. Patients were excluded if they had a severe or unstable medical disease that could interfere with the successful completion of the study, a clinically significant laboratory abnormality, abnormally low levels of vitamin B12 or folate, positive syphilis serology, a primary neurodegenerative other than AD, or drug or alcohol addiction during the past 10 years. The severity of Alzheimer’s disease was classified as mild (MMSE score 21~26), moderate (MMSE score 10~20), moderately severe (MMSE score 10~14) and severe (MMSE score below 10). Control individuals were consisted of elderly people (age ≥ 60) without cognitive symptoms (MMSE score ≥ 24).

Whole blood (10 ml) was collected from participants in EDTA-containing Vacutaner tubes (Becton Dickinson, 366643). Collected whole blood was stored in 300-μl aliquots at −80°C until use.

### RNA extraction and quantitative reverse-transcription polymerase chain reaction (qRT- PCR)

Total RNA was extracted using the RNeasy Plus Mini Kit (Qiagen, #74134) and RNeasy Lipid Tissue Mini Kit (Qiagen, #74804) for cultured cells and brain tissue, respectively, according to the manufacturer’s protocols. Total RNA (1 μg) was converted to cDNA using Superscript II reverse transcriptase (Invitrogen, 18064–014) and oligo-(dT)_12-18_ primers (Bioline, Bio-38029) according to the manufacturer’s instructions. qRT-PCR was performed in a 20-μl reaction mixture containing 1 μl cDNA, 10 μl SYBR Premix EX Taq (Takara Bio, RR420A), 0.4 μl Rox reference dye (50×, Takara Bio, RR420A), and 200 nM primers for each gene. The primer sequences were as follows: human *HMOX1* (forward), 5′-GGAACTTTCAGAAGGGCCAG-3′; human *HMOX1* (reverse), 5′- GGAAGTAGACAGGGGCGAAG-3′; mouse *HMOX1* (forward), 5′- CACTTCGTCAGAGGCCTGCTA-3′; mouse *HMOX1* (reverse), 5′- GTCTGGGATGAGCTAGTGCTGAT-3′; human *GAPDH* (forward), 5′-AATCCCATCACCATCTTCCA-3′; human *GAPDH* (reverse), 5′-TGGACTCCACGACGTACTCA-3′; mouse *GAPDH* (forward), 5′-AATGTGTCCGTCGTGGATCT-3′; mouse *GAPDH* (reverse), 5′-GGTCCTCAGTGTAGCCCAAG-3′. Reactions were run on an ABI PRISM 7000 sequence detection system (Applied BioSystems) at 50°C for 2 min and 95°C for 10 min followed by 40 cycles of 95°C for 15 sec and 60°C for 1 min and a dissociation stage of 1 cycle at 95°C for 15 sec, 60°C for 20 sec, and 95°C for 15 sec. All PCR reactions were performed in triplicate, and the specificity of the reaction was detected by melting curve analyses at the dissociation stage. Comparative quantification of each target gene was performed based on the cycle threshold (C_T_), which was normalized to *GAPDH* using the ΔΔC_T_ method.

### Transcriptome sequencing analysis

Total RNA (5 μg) was subjected to two rounds of hybridization to oligo(dT) beads (Invitrogen, #610.02). The resulting mRNA was then used as a template for cDNA synthesis. mRNA was randomly fragmented between 200 and 700 bp by focused acoustic shearing (Covaris Inc.) and converted to first-strand cDNA using Superscript III (Invitrogen, #18080–051) followed by second-strand cDNA synthesis using Escherichia coli DNA polymerase I (Invitrogen, #18012–021). The double-stranded cDNA library was further processed by Illumina Genomic DNA Sample Prep Kit (Illumina Inc., FC-102-1001), which involved end repair using T4 DNA polymerase (Invitrogen, EP0061), Klenow DNA polymerase (Invitrogen, #18012–039), and T4 Polynucleotide kinase (Invitrogen, AM2310) followed by a single <A> base addition using Klenow 3' to 5' exo-polymerase (Invitrogen, #EP0421) and ligation with Illumina's adaptor oligo mix (Illumina Inc., #PE400-1001) using T4 DNA ligase (Invitrogen, 15224–041). The adaptor-ligated library was size-selected by separating on a 2% agarose gel and cutting out the library smear at 500 bp. The library was PCR-amplified for 18 cycles using Phusion DNA Polymerase (New England BioLabs, M0530S) and purified using the Qiaquick PCR Purification Kit (Qiagen, #28104). The library was quantified using the Quant-iT picogreen dsDNA Assay Kit (Invitrogen, P11496) following the manufacturer's protocol. We prepared Genome Analyser paired-end flow cell on the supplied Illumina cluster station (Illumina Inc.) and generated 38-bp paired-end sequence reads on the Illumina Genome Analyser *IIx* platform (Illumina Inc.) following the manufacturer's protocol. Primary data analysis including image analysis, base-calling, and alignment, which was carried out with the Illumina pipeline. The RNA-sequencing read files have been deposited in NCBI Bioproject under accession number SRP062936.

### Western blot analyses

Proteins (40–50 μg) were resolved using denaturing 10–12% sodium dodecyl sulfate- polyacrylamide gel electrophoresis and transferred to polyvinylidene fluoride membranes. Membranes were blocked in 5% skim milk in Tris-buffered saline with 0.1% Tween 20 and incubated overnight at 4°C with the following primary antibodies: mouse anti-HMOX1 polyclonal antibody (1:1,000, Sigma-Aldrich, SAB14059), mouse anti-HMOX1 monoclonal antibody (1:250, abcam, ab13248) or mouse anti-β-actin monoclonal antibody (1:2,000, Santa Cruz Biotechnology, sc-47778). After washing, membranes were incubated with secondary antibodies conjugated to horseradish peroxidase for 1 h at room temperature. Chemiluminescence was detected using peroxidase substrate (Ab frontier, LF-QC0106) according to the manufacturer's protocol. Bands were visualized using a Luminescent Image analyser LAS-300 (General Electric) and quantified using Image Gauge software (FujiFilm).

### Genomic DNA extraction and DNA modification using bisulfite

Genomic DNA was isolated from the cell line and whole blood using a QIAamp DNA Mini Kit (Qiagen, #51306). Treatment of the extracted DNA with bisulfite converts unmethylated cytosine but not methylated cytosine to uracil, allowing for the distinction between unmethylated and methylated cytosine. DNA modification using bisulfite was performed using an EpiTech Bisulfite Kit (Qiagen, #59104) according to the manufacturer’s manual.

### Methylation microarray

For genome-wide screening of DNA methylation, the Illumina HumanMethylation27 BeadChip (Illumina Inc., WG-311-2201) targeting 27,578 specific CpG sites located within the proximal promoter regions of transcription start sites of 14,475 consensus coding sequences in the National Center for Biotechnology Information database was used according to the manufacturer’s recommendations. DNA methylation values were described as β-values, which were calculated by subtracting the background of negative controls on the array and then computing the ratio of methylated signal intensity to the sum of both methylated and unmethylated signal intensities. β-values ranged from 0 (completely unmethylated) to 1 (fully methylated) on a continuous scale for each CpG site. Forty-three targets with detection *p*-values >0.05 were excluded from the 27,578 targets; thus, a total of 27,535 CpG targets were used in the final analysis. To identify differentially methylated CpG sites in H4 and H4-sw cell lines, we considered differences in mean β-values (Δβ; mean β-value in H4-sw–mean β-value in H4). If the absolute difference in mean β-values (|Δβ|) between the two cell lines was >0.06 (i.e., greater than the standard deviation at identical CpG sites), the site was defined as a differentially methylated CpG site. We considered hypermethylated CpG sites/genes as those with Δβ greater than 0.06 and hypomethylated CpG sites/genes as those with Δβ less than −0.06. The primary CpG methylation values have been deposited in NCBI Gene Expression Omnibus _under accession number GSE72538.

### Quantitative methylation-specific PCR (qMSP)

To measure the methylation level of particular CpG sites in the *HMOX1* gene promoter, genomic DNA was treated with bisulfite, and then each primer was used to distinguish methylated DNA from unmethylated DNA at the particular CpG sites. The primer sequences used were as follows: methylated *HMOX1* (forward), 5’-TTATTAGGTTATTGTTTTGAGTAGC-3’; unmethylated *HMOX1* (forward), 5’-TTATTAGGTTATTGTTTTGAGTAGT-3’; methylated/unmethylated *HMOX1* (reverse) 5’-TCCCAAAAAATTCCAAAAAACTAAA-3’; internal control *HMOX1*, 5’-AGTAGGTGATATTTTAGGGAGT-3’ (forward); internal control *HMOX1*, 5’-TCCCAAAAAATTCCAAAAAACTAAA-3’ (reverse).

For qMSP, 20 μl reaction mixture containing 2 μl (10–100 ng/μl) bisulfite-treated DNA, 10 μl SYBR Premix EX Taq (Takara Bio, RR420A), 0.4 μl Rox reference dye (50×; Takara Bio, RR420A), and 200 nM of each primer was reacted using a 7500 Fast Real-time PCR system (Applied Biosystems) at 95°C for 30 sec followed by 40 cycles of 95°C for 3 sec and 62°C for 30 sec for amplification. The PCR product was reacted at 95°C for 15 sec, 60°C for 1 min, and 95°C for 15 sec to examine specificity. Methylation and unmethylation of the specific CpG sites were normalized using an internal control, and calculated as follows (Ct represents the threshold cycle):

$$\Delta {Ct}_{meth}\;=\;{Ct}_{meth}-{Ct}_{internal}$$

$$\Delta {Ct}_{unmeth}\;=\;{Ct}_{unmeth}-{Ct}_{internal}$$

$$\%\;methylation\;=\;\frac{100}{1+2^{\left({\Delta Ct}_{meth}-{\Delta Ct}_{unmeth}\right)}}$$

### Bisulfite sequencing analyses of DNA methylation patterns using the 454 GS-FLX system

Bisulfite PCR of the *HMOX1* promoter regions was performed in 50 μl reactions containing 10 ng bisulfite-modified genomic DNA, 1.5 mM MgCl_2_, 200 μM dNTPs, 1 U Platinum Taq polymerase (Invitrogen, #10966018), 1× Platinum Taq buffer, and 200 nM each specific BSP forward and reverse primers. The BSP primers were designed using MethPrimer software (www.urogene.org/methprimer/). The bisulfite *HMOX1* PCR product was 465 bp (position in UCSC gene database human GRCh38/hg38, chromosome 22, 35,380,564–35,380,907) and contained 9 CpG sites. Sequences of the bisulfite PCR primers are: 5′- TAAAGAGGGTGTGAGGAGGT-3′ (forward) and 5′-ACAACTAATACCCACTTTCTAA-3′ (reverse). The reaction was run at 95°C for 5 min, followed by 30 cycles at 95°C for 30 sec, 55°C for 30 sec, and 72°C for 30 sec. There was a final elongation step at 72°C for 5 min. Bisulfite PCR products were purified using QIAquick Gel Extraction kits (Qiagen, #28704) according to the manufacturer’s protocol.

A library was prepared according to the GS FLX titanium library prep guide using bisulfite PCR products. Libraries were quantified using Ribogreen assays (Invitrogen, R11490). The emPCR, corresponding to clonal amplification of the purified library, was performed using the GSFLX titanium emPCR Kit (Roche/454 Life Sciences, #05233542001). Briefly, libraries were immobilized onto DNA capture beads. The library-beads obtained were added to a mixture of amplification mix and oil, and vigorously shaken on a Tissue LyserII (Qiagen) to create "micro-reactors" containing both amplification mix and a single bead. The emulsion was dispensed into 96-well plates and the PCR amplification program was run according to the manufacturer's recommendations. Following amplification, the emulsion was chemically broken and beads carrying the amplified DNA library were recovered and washed by filtration. Positive beads were purified using the biotinylated primer A (complementary to adaptor A), which binds streptavidin-coated magnetic beads. DNA library beads were then separated from magnetic beads by melting the double-stranded amplification products, leaving a population of bead-bound single-stranded template DNA fragments. The sequencing primer was then annealed to the amplified single-stranded DNA. Finally, beads carrying amplified single-stranded DNA were counted using a Particle Counter (Beckman Coulter). Sequencing was performed on a Genome Sequencer FLX titanium (Roche/454 Life Sciences), and each sample was loaded in one region of a 70 mm × 75 mm Pico Titer plate (Roche/454 Life Sciences) fitted with an 8-lane gasket.

For data analyses, we used Amplicon Variant Analyzer (AVA) software (Roche). The report includes auto-detected variants using the "computation load detected variants" command from the command line interface (CLI). The auto-detected variants show the frequency at which all the variants defined in the project were observed.

### Bisulfite sequencing PCR (BSP)

Genomic DNA of H4 and H4-sw cell lines were subjected to BSP to identify CpG sites of the *HMOX1* gene promoter with differences in methylation status. Primers capable of amplifying the particular promoter region of *HMOX1* (UCSC gene database human GRCh38/hg38, chromosome 22, 35,380,082–35,381,037) were prepared and used to amplify bisulfite-treated DNAs. BSP was carried out using conventional PCR in a 50 μl reaction mixture containing 10 ng bisulfite-modified genomic DNA, 1.5 mM MgCl_2_, 200 μM dNTP, 1 U Platinum Taq polymerase (Invitrogen, 11306–016), 1× Platinum Taq buffer, and 200 nM specific BSP forward and reverse primers for each gene. BSP primers were designed using MethPrimer software (http://www.urogene.org/methprimer). For *HMOX1*, the BSP product was 956 bp and contained 17 CpG sites. The BSP primer sequences were: (forward), 5’-TAAAGAGGGTGTGAGGAGGT-3’; and (reverse), 5’-ACAACTAATACCCACTTTCTAA-3’. The reaction ran at 95°C for 5 min followed by 30 cycles of 95°C for 30 sec, 50–55°C for 30 sec, and 72°C for 30 sec and a final elongation step at 72°C for 5 min. BSP products were purified using the QIAquick Gel Extraction kit (Qiagen, #28704) according to the manufacturer’s protocol and ligated into the pGEM-T Easy cloning vector (Promega, A1360). The ligation products were used to transform competent DH5α *Escherichia coli* cells (RBC Bioscience, RH618) using standard procedures. Blue/white screening was used to select bacterial clones, and BSP product-positive clones were confirmed by colony PCR using the BSP primers to verify insert size. Plasmid DNA was then extracted from at least 10 insert-positive clones using the QIAprep Spin Miniprep Kit (Qiagen, #27104) and sequenced using M13 primer to analyze methylation status at specific CpG sites.

### 5-aza-2´-deoxycytidine treatment

H4 cells were treated with the methylation inhibitor 5-aza-2´-deoxycytidine (Sigma-Aldrich, A3656), which was replaced daily at a concentration of 10 μM for 3 days. Inhibition of methylation was examined by qMSP, and changes in *HMOX1* gene expression were measured by qRT-PCR.

### Amyloid-β (Aβ_1–42_ or Aβ_1–40_) preparation and treatment

Synthetic Aβ_1–42_ or Aβ_1–40_ (American Peptide Company, 62-0-80 or 62-0-65) was dissolved in hexafluoro-2-propanol (HFIP, Sigma-Aldrich, #105228) for 72 h in the dark and aliquoted into microcentrifuge tubes. HFIP was removed by evaporation in a vacuum concentrator (Hanil Science Industrial). The tubes were stored at −80°C until use. To prepare a soluble form of Aβ_1–42_ or Aβ_1–40_, dried Aβ_1–42_ or Aβ_1–40_ was dissolved in dimethylsulfoxide (Sigma-Aldrich, #276855) to make a 1 mM stock solution. The Aβ_1–42_ or Aβ_1–40_ stock solution was immediately diluted in serum-free media to make final 1 μM working solutions. H4 cells were treated with soluble Aβ_1–42_ or Aβ_1–40_ at 1 μM for 5 days. The media containing Aβ_1–42_ or Aβ_1–40_ was replaced daily.

The human leukemia cells were treated with oligomeric form of Aβ_1–42_. To prepare the oligomeric form of Aβ_1–42_, the soluble stock solution was first diluted in phosphate-buffered saline (PBS) to a concentration of 10 μM, mixed vigorously, and incubated at 4°C for 24 h as previously described [32]. The oligomeric form of Aβ_1–42_ was further diluted in culture media containing 1% FBS to make final 1 μM working solutions. Jurkat, U937, HL60 and THP-1 cells were treated with 1 μM oligomeric form of Aβ_1–42_ for 6 days. The media containing the oligomeric form of Aβ_1–42_ was replaced daily.

### Statistical analysis

All data are expressed as mean±standard deviation (SD) of at least three independent experiments. Statistical analyses were carried out using GraphPad Prism 5 software. The details of each statistical analysis are provided in the figure legends. *P* values < 0.05 were considered statistically significant.

## Results

### Up-regulation of HMOX1 gene expression in a cellular AD model

**Fig 1:**
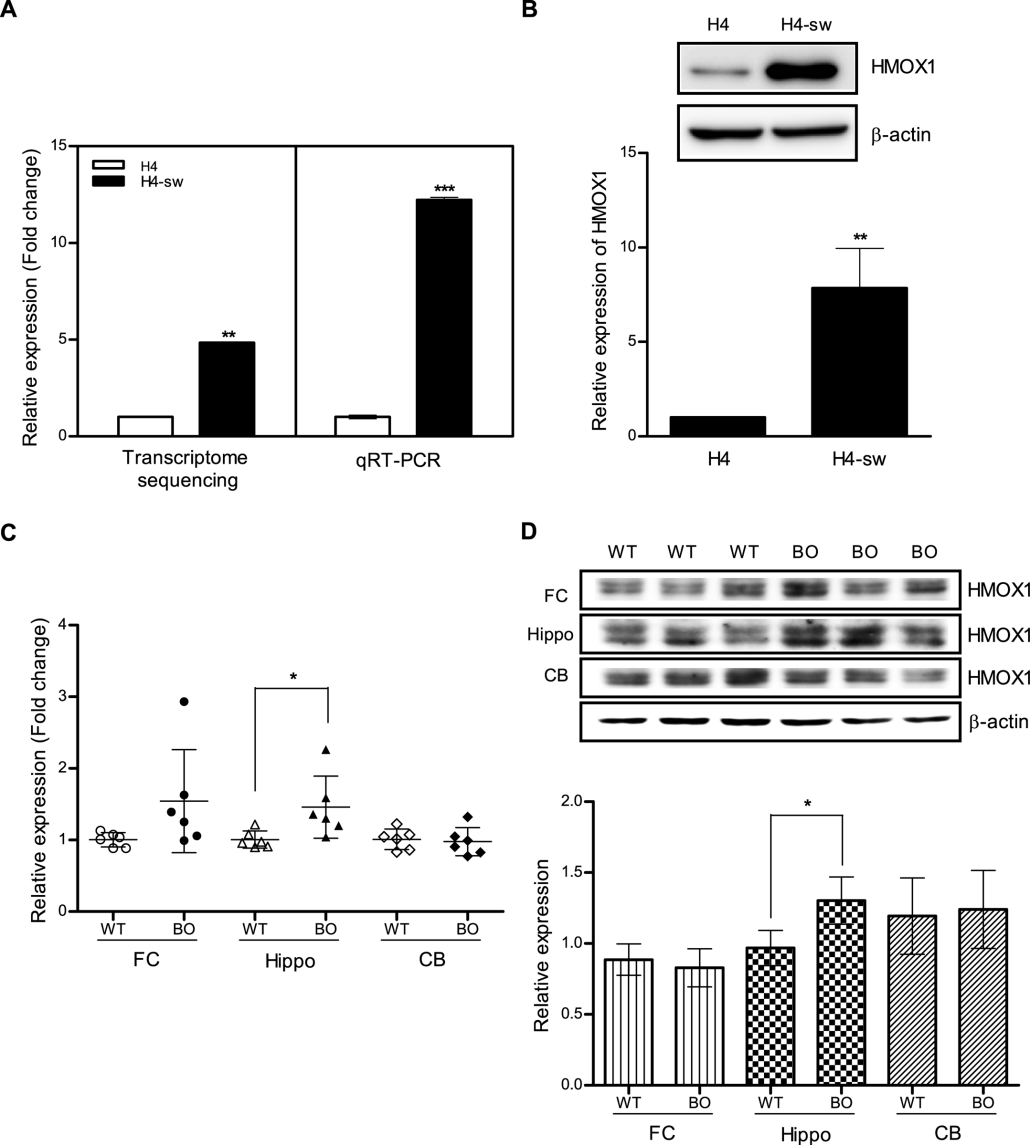
HMOX1 expression is up-regulated in an APP-mutant cell line and transgenic mice. (A) *HMOX1* mRNA expression in H4 and H4-sw cells was measured by transcriptome sequencing analyses and qRT-PCR. (B) Expression of HMOX1 protein in H4 and H4-sw cells was assessed using western blot analyses. Level of HMOX1 protein in H4-sw cells was expressed relative to that in H4 cells. (C) *HMOX1* mRNA expression in the brain of 12-month-old wild-type and PSEN1-APP transgenic mice (B6C3-Tg(APP695)85Dbo Tg(PSEN1)85Dbo) was measured by qRT-PCR. (D) HMOX1 protein expression in the brain of 12-month-old wild-type and PSEN1-APP transgenic mice assessed using western blot analyses. Data are shown as mean ± SD. Statistical analyses were performed using *t*-tests (* p < 0.05, ** p < 0.01, *** p < 0.001). H4-sw, APP-Swedish mutant H4 cells; FC, frontal cortex; Hippo, hippocampus; CB, cerebellum; WT, wild-type; BO, PSEN1-APP transgenic mice.

We used H4-sw cells, which show elevated levels of toxic Aβ_1–42_ and phosphorylated tau protein [33], as a cellular AD model. Previously, we have reported global gene expression profiles that 283 genes were down-regulated and 348 genes were up-regulated in H4-sw cells compared wild type H4 cells with the criteria of a fold change greater than 2, transcript number larger than 3 and p value less than 0.05 [34]. Among these genes, *HMOX1* expression was up-regulated approximately 4.8-fold in H4-sw cells (Fig 1A). Consistent with our transcriptome sequencing data, *HMOX1* mRNA levels as well as protein levels were approximately 12.2-fold and 7.9-fold increased in H4-sw cells, respectively, compared to wild type H4 cells (Fig 1A and 1B). Increased *HMOX1* mRNA expression and protein expression were also observed in the hippocampus of 12-month-old APP swe/PSEN1 transgenic mice (Borchelt; B6C3-Tg(APP695)85Dbo/Tg(PSEN1)85Dbo) compared with wild-type mice, although *HMOX1* mRNA and protein levels remained unchanged in the frontal cortex and cerebellum of transgenic mice (Fig 1C and 1D).

### Regulation of HMOX1 expression by DNA methylation

**Fig 2:**
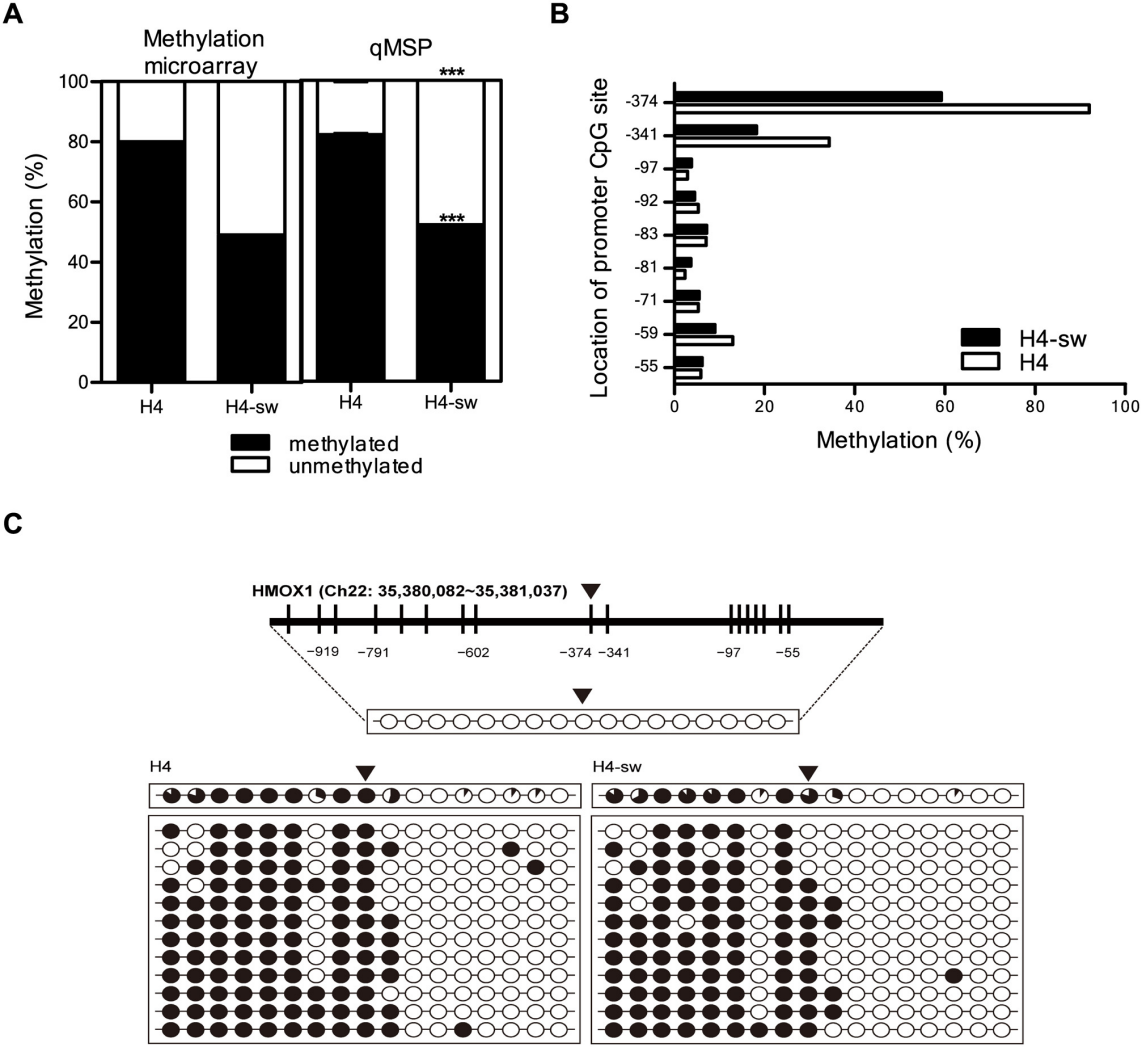
Hypomethylation of specific CpG sites within the *HMOX1* promoter in H4-sw cells. DNA methylation status at the −374 CpG site was analyzed using (A) the Illumina HumanMethylation 27 BeadChip, (A) qMSP and (B) the 454 GS-FLX system. (C) The *HMOX1* promoter region is located at position 35,380,082–35,381,037 in the human GRCh38/hg38 assembly and contains 17 CpG residues within chromosome 22. The 17 CpGs are located at positions −959, −919, −876, −791, −743, −706, −628, −602, −374, −341, −97, −92, −83, −81, −71, −59, and −55 from the transcription start site. Each circle represents CpG dinucleotides. The methylation status of each CpG site is illustrated by black (methylated) and white (unmethylated) circles, and the total percentage of methylation at each site is indicated by a pie graph on the top line. The black area of the pie graph indicates the methylated CpG percentage, whereas the white area indicates the unmethylated CpG percentage. Triangles above the circles or the bar in C indicate the specific CpG site that was used for qMSP and methylation microarray. Statistical analyses were performed using *t*-tests (*** p < 0.001). H4-sw, APP-Swedish mutant H4 cells.

To investigate epigenetic abnormalities in AD, we performed global DNA methylation profiling on H4 and H4-sw cells using the human methylation 27 bead chip [35]. We found that DNA methylation of the CpG site at position −374 from the *HMOX1* transcription start site was markedly decreased in H4-sw cells compared with H4 cells, and this hypomethylation was confirmed by methylation-specific PCR (Fig 2A). We further examined DNA methylation of the *HMOX1* promoter in H4 and H4-sw cells using bisulfite sequencing analyses to identify critical CpG sites. The *HMOX1* promoter region (chromosome 22, 35,380,564–35,380,907 in human GRCh38/hg38) was PCR amplified using bisulfide-modified genomic DNA as a template to obtain 465 bp bisulfite PCR products in H4 and H4-sw cells. DNA methylation patterns of PCR amplicons were analyzed using the 454 GS-FLX system. Our results show that promoter CpGs at −374 and −341 within the PCR amplicon were significantly hypomethylated in H4-sw cells compared to H4 cells (Fig 2B). We also tested longer *HMOX1* promoter region contained 17 CpG sites located at positions −959, −919, −876, −791, −743, −706, −628, −602, −374, −341, −97, −92, −83, −81, −71, −59, and −55 from the transcription start site using BSP (Fig 2C). We observed that 10 of the 17 CpG sites were hypomethylated in H4-sw cells compared with H4 cells, with particularly severe hypomethylation observed at −374 and −341 CpG sites in agreement with the result of the 454 GS-FLX system (Fig 2C).

**Fig 3:**
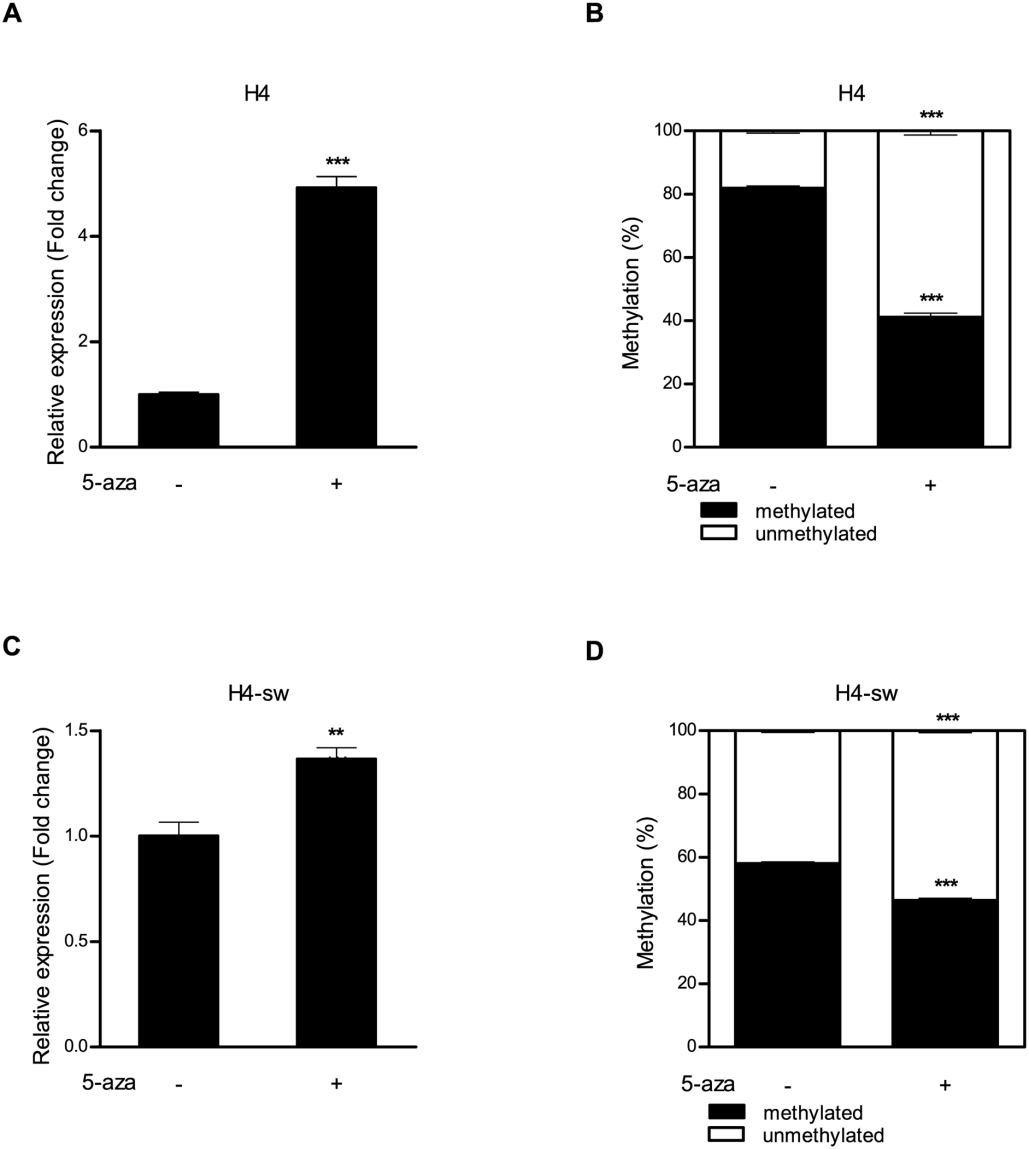
Changes in HMOX1 expression following demethylation in H4 and H4-sw cells. H4 and H4-sw cells were treated with 10 μM 5-aza-2′-deoxycytidine for 3 days. After treatment, (A and C) *HMOX1* mRNA expression was measured by qRT-PCR. (B and D) DNA methylation status at the −374 CpG site was measured using qMSP. Data are shown as mean ± SD (n = 3). Statistical analyses were performed using *t*-tests (** p < 0.01, *** p < 0.001). 5-aza, 5-aza-2′-deoxycytidine.

Next, we determined whether *HMOX1* gene expression is controlled by an epigenetic regulatory mechanism. After H4 cells were treated with the demethylating agent 5-aza-2´-deoxycytidine at a concentration of 10 μM for 3 days, the expression of *HMOX1* mRNA was significantly increased (Fig 3A), and decreased methylation status at the −374 CpG site was confirmed using qMSP (Fig 3B). Similar to the results of H4 cells, increased expression of *HMOX1* mRNA and decreased methylation status at the −374 CpG site were observed in H4-sw cells. However, alteration of the mRNA expression and the methylation status after treatment with 5-aza-2´-deoxycytidine in H4-sw cells were much smaller than those of H4 cells (Fig 3C and 3D).

### Epigenetic regulation of HMOX1 by Aβ_1–42_ or Aβ_1–40_

**Fig 4:**
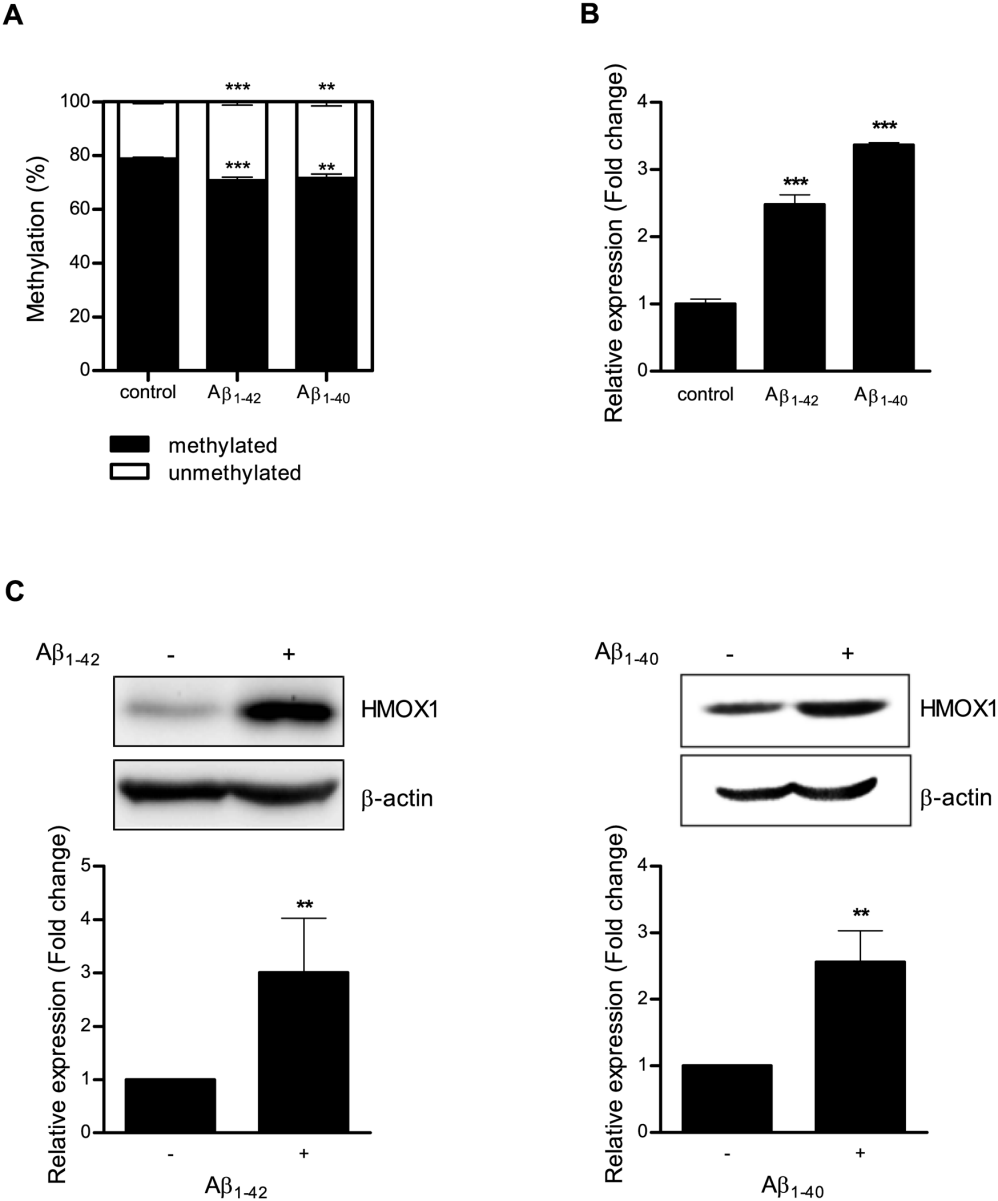
DNA methylation is altered in H4 cells treated with Aβ_1–42_ or Aβ_1–40._ (A) H4 cells were treated with 1 μM Aβ_1–42_ or Aβ_1–40_ for 5 days, and DNA methylation at the –374 CpG site was analyzed using qMSP. (B) After treatment with Aβ_1–42_ or Aβ_1–40,_ *HMOX1* expression was assessed using qPCR. (C) Protein levels of HMOX1 were determined by western blot analyses. Values shown are relative to untreated controls. Data are shown as mean ± SD (n = 3). Statistical analyses were performed using *t*-tests (** p < 0.01, *** p < 0.001).

After Aβ_1–42_ treatment at a concentration of 1 μM for 5 days, DNA methylation status at the −374 CpG site was determined using qMSP. We found that DNA methylation at the −374 CpG site of *HMOX1* was decreased in treated H4 cells compared with untreated H4 cells (Fig 4A). Furthermore, *HMOX1* mRNA and protein expression were significantly increased in treated H4 cells (Fig 4B and 4C). Similar to the results of Aβ_1–42_ treatment, after H4 cells were treated with Aβ_1–40_ for 5 days, *HMOX1* mRNA and protein expression were significantly increased whereas DNA methylation at the −374 CpG site of *HMOX1* was decreased in Aβ_1–40_-treated H4 cells compared with untreated H4 cells (Fig 4A–4C). These results show that not only Aβ_1–42_ but also Aβ_1–40_ induce aberrant epigenetic modifications in the *HMOX1* promoter region and subsequently up-regulates *HMOX1* expression.

**Fig 5:**
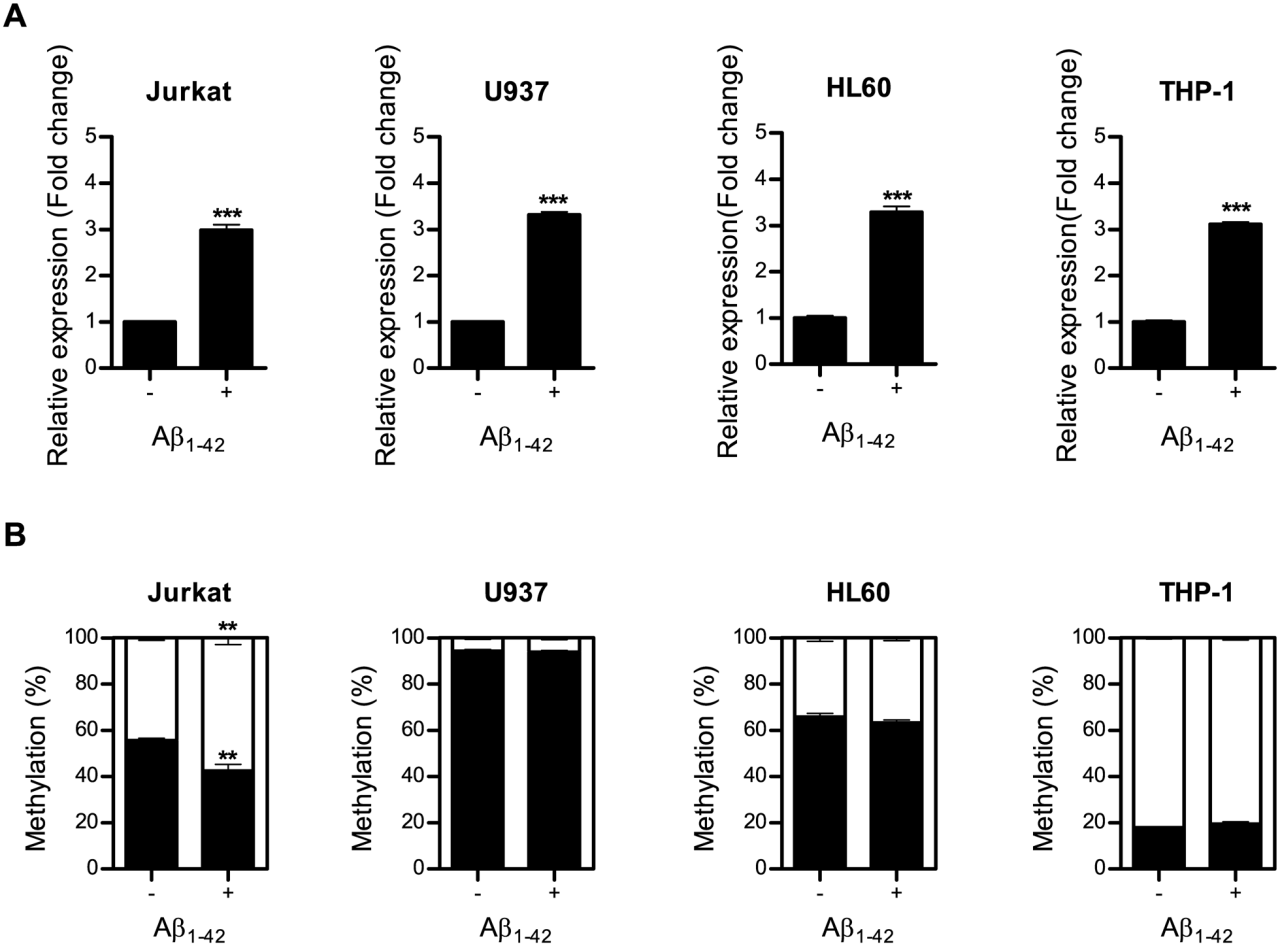
Alterations of DNA methylation induced by Aβ are cell-types specific. Human leukemia cells (Jurkat, U937, HL60 and THP-1) were treated with 1 μM Aβ_1–42_ for 6 days. After treatment with Aβ_1–42_ (A) *HMOX1* mRNA expression was assessed using qPCR, (B) DNA methylation of *HMOX1* at the –374 CpG site was analyzed using qMSP. Values shown are relative to untreated controls. Data are shown as mean ± SD (n = 3). Statistical analyses were performed using *t*-tests (** p < 0.01, *** p < 0.001).

To investigate whether Aβ-induced epigenetic regulation of *HMOX1* could be occurred in other cell types, especially in blood-derived cells, we treated human leukemia cells (Jurkat, U937, HL60 and THP-1) with 1 μM oligomeric form of Aβ_1–42_ for 6 days and then determined DNA methylation level of *HMOX1* at the −374 CpG site and the expression of HMOX1 before and after treatment. Previous study has been reported more robust correlation between plasma oligomeric Aβ level and cognition of AD patients than other forms of Aβ, we therefore used oligomeric form of Aβ_1–42_ to treat human leukemia cells [36]. Although the expression of HMOX1 was up-regulated in all types of blood-derived cells (Fig 5A), Aβ-induced epigenetic regulation of *HMOX1* was only found in Jurkat cells (Fig 5B) showing Aβ-induced epigenetic regulations are cell-types specific.

### Close relationship between blood *HMOX1* gene methylation status and cognitive impairment in AD patients

**Fig 6:**
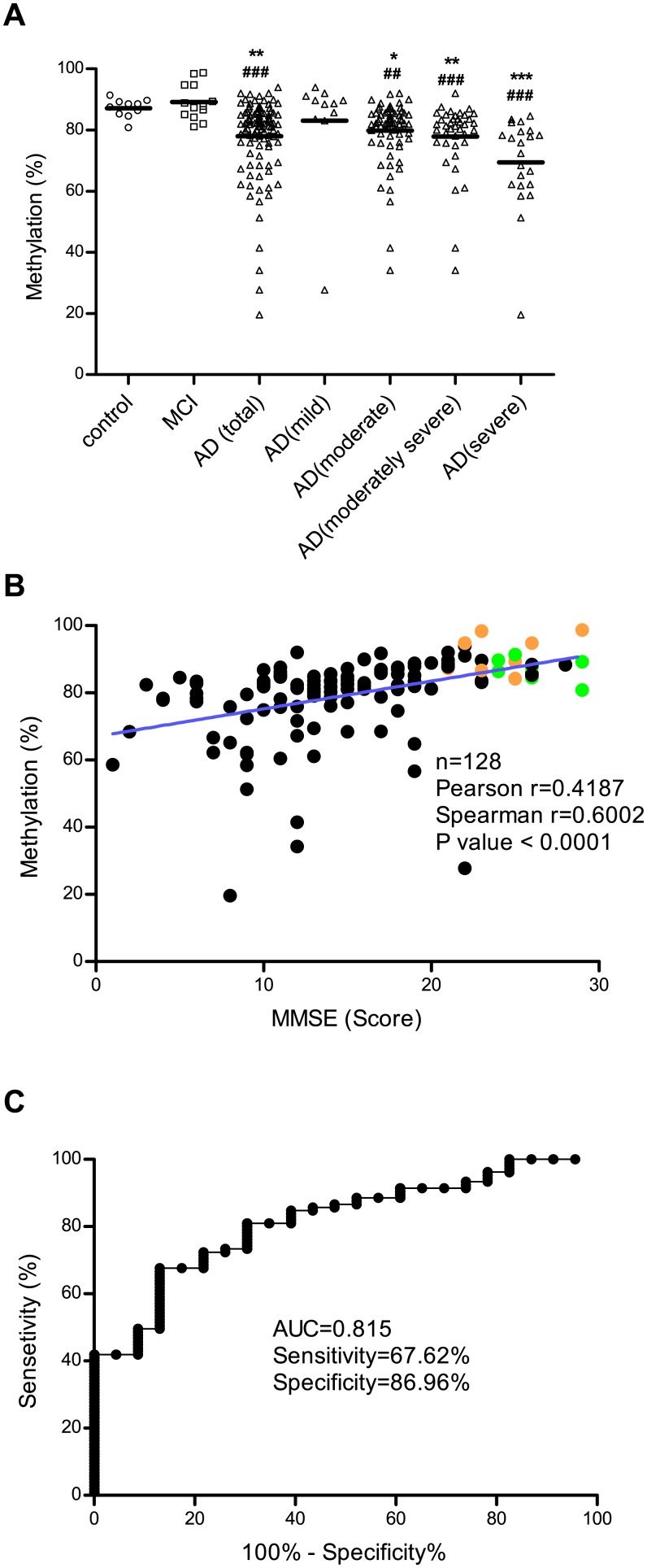
Evaluation of DNA methylation status at the –374 CpG site on the *HMOX1* gene as a biomarker for AD. DNA methylation at the –374 CpG site in blood samples from AD patients, MCI patients and control individuals was analyzed using qMSP. (A) Data are expressed as scattered plots with lines at mean value. Statistical analyses were performed using Kruskal-Wallis one-way analysis of variance and Dunn’s multiple comparison post-tests for comparing the significance between AD patients and control individuals (*) or MCI patients (^#^) (*, ^#^ *p* < 0.05, **, ^##^ p < 0.01, ***, ^###^ p < 0.001). (B) The relationship between DNA methylation at the –374 CpG site on the *HMOX1* gene and MMSE score was evaluated by Pearson and Spearman correlations (green dots, control individuals; orange dots, MCI patients; black dots, AD patients). (C) Receiver operating characteristic curves shows the discrimination of AD patients from MCI patients and control individuals based on DNA methylation status at the –374 CpG site of *HMOX1*. The area under the curve is 81.50% for AD.

Genomic DNA was extracted from whole blood of AD patients, MCI patients, and control individuals, and the methylation status of the −374 CpG site on the *HMOX1* gene was examined using qMSP. The study population characteristics are summarized in Table 1 and are described more in detail in S1 Table. We found that AD patients had significantly lower methylation levels (mean ± SD; 77.94 ± 12.97) than MCI patients (89.15 ± 5.85) and control individuals (87.20 ± 3.06), but there was no difference in methylation status between MCI patients and control individuals (Fig 6A). The power of detecting a significant mean difference in methylation (%) in all pairs of AD, MCI and control groups at the significance level of 0.05 using a t-test were 84% (AD and control), 96% (AD and MCI) and 16% (MCI and control). This indicates that our study had power to detect differences between AD and control/MCI groups although the power was lack to detect the difference between MCI and control groups (S1 Fig).

**Table 1:** Subject demographics.

Characteristics	AD, n = 105	MCI, n = 13	Control, n = 10
**Gender (female)**			
No. (%)	79 (75)	6 (46)	2 (20)
**Age, yr**			
Mean (SD)	81.26 (5.44)	73.62 (6.19)	72.10 (7.40)
Range	64~93	58~80	60~84
**MMSE score**			
Mean (SD)	13.71(5.32)	22.15 (3.56)	26.10 (1.73)
Range	1~28	16~26	24~29
**APOE ε4 carriers**^a^			
No. (%)	24 (26)	4 (31)	3 (30)

Abbreviations: AD, Alzheimer’s disease; MCI, mild cognitive impairment; MMSE, mini-mental state examination.

^a^APOE information from 14 patients of AD is missing.

The severity of Alzheimer’s disease was classified as mild (MMSE score 21~26), moderate (MMSE score 10~20), moderately severe (MMSE score 10~14) and severe (MMSE score below 10). The scattered plots showed that lower methylation levels were associated with more severe disease in patients with AD (Fig 6A).

The relationship between DNA methylation at the −374 CpG site on the *HMOX1* gene and MMSE score was evaluated by Pearson and Spearman correlation analyses. We found that lower MMSE scores were associated with lower levels of methylation at the −374 CpG site in all individuals (Fig 6B). For the methylation level of HMOX1, gender and APOE ε4 genotype were not found to be statistically significant factors (S2 Fig) whereas age was inversely correlated with the methylation of HMOX1 (S3 Fig). Although age was significantly correlated with both MMSE (Pearson correlation r = −0.589, p < 0.001) and methylation level of HMOX1 (Pearson correlation r = −0.465, p < 0.001) as shown in S3 Fig, the correlation between methylation level of HMOX1 and MMSE was still significant (partial correlation coefficient: 0.203, p = 0.022) even after adjustment for age. Receiver operating characteristic analysis revealed good separation between AD patients and the other two groups (MCI patients and control individuals) with a sensitivity of 67.62% and a specificity of 86.96% (Fig 6C). Taken together, these results suggest that the methylation level of a particular promoter region of *HMOX1* (i.e., the CpG site located −374 from the transcription start site) can be used as a diagnostic biomarker for predicting AD progression.

## Discussion

HMOX1 is an enzyme that mediates the degradation of heme to Fe^2+^, CO, and biliverdin/bilirubin. Previous studies show that HMOX1 expression is up-regulated in cortical and hippocampal neurons and astrocytes in patients with AD or MCI, whereas its expression is faint or undetectable in age-matched normal tissue [14, 37]. Consistent with these previous findings, our results show that *HMOX1* mRNA and protein expression was dramatically increased in H4-sw cells. Similarly, increased *HMOX1* expression was detected in the brain, particularly the hippocampus, of APP swe/PSEN1 transgenic mice. To explore the expression patterns of HMOX1 in AD-affected brain regions, we analyzed mRNA expression levels of HMOX1 for multiple brain regions of control and AD patients using publically available databases, GSE5281 and GSE15222. The results of analyses revealed that in late onset AD patients, mRNA expressions of HMOX1 were increased in AD-affected brain regions but significant increase was only observed in the cortex rather than hippocampus where we have shown the significant increase of HMOX1 in APP swe/PSEN1 transgenic mice. This discrepancy may reflect the differences in pathogenic development between familial versus sporadic forms of AD, and/or differences in disease progression between disease-progressing mouse brain versus postmortem human brain (S4 Fig).

*HMOX1* expression is induced by various stimuli including reactive oxygen species, reactive nitrogen species, lipopolysaccharides, ischemia, heatshock, hemin, and Aβ [12, 38]. The induction of *HMOX1* is transcriptionally regulated by heterodimers of Maf proteins with NF-E2-related factor2 or activator protein 1, and the inhibition of its expression is finely regulated through the transcriptional repressor Bach-1 [13, 38]. In addition to regulation by transcription factors, the present findings also demonstrate a novel regulatory mechanism of *HMOX1* expression. We found that *HMOX1* expression was aberrantly up-regulated in H4-sw cells, and the methylation of specific CpG sites within its promoter, particularly at CpGs located −374 and −341 from the transcriptional start site, was lower in H4-sw cells than in H4 cells. Moreover, treatment of H4 and H4-sw cells with the DNA methyltransferase inhibitor 5-aza-2’-deoxycytidine enhanced *HMOX1* expression. These results suggest that the transcriptional regulation of *HMOX1* expression is controlled by DNA methylation-dependent epigenetic regulation.

Previous studies report that aberrant epigenetic alterations such as global hypomethylation or hypermethylation of certain genes can be induced by toxic Aβ and may contribute to the pathogenesis of AD [32, 39]. Our results confirm that abnormal epigenetic alterations of *HMOX1* can be mediated by toxic Aβ. Specifically, promoter CpG methylation located at −374 from the transcriptional start site was decreased in H4 cells treated with Aβ_1–42_ or Aβ_1–40_, and methylation status was correlated with increased *HMOX1* mRNA and protein expression. These findings suggest that toxic Aβ-induced aberrant DNA methylation could be an important regulatory mechanism that controls expression of *HMOX1* in AD pathogenesis. In addition to glioblastoma cells, we also found Aβ-induced epigenetic regulation of *HMOX1* in human T lymphocyte Jurkat cells suggesting epigenetic modification of *HMOX1* in certain type of blood cells may reflect regulatory mechanisms observed in neural cells.

We detected less DNA methylation of the *HMOX1* gene in the blood of AD patients compared with MCI patients and control individuals, and reduced levels of DNA methylation of the *HMOX1* gene at the −374 CpG site were closely associated with cognitive decline in AD patients. Although both DNA methylation level of HMOX1 and age were significantly associated with cognition, the correlation between methylation level of HMOX1 and MMSE score was significant even after adjustment for age. This result suggests that toxic Aβ induced aberrant epigenetic alteration in the HMOX1 promoter region may cause the chronic overexpression of HMOX1 and reflect memory decline of AD patients. Recently, a link between HMOX1 overexpression and cognitive deficits in AD was reported by Luo et al., who found that the long-term overexpression of HMOX1 induces memory decline in transgenic mice through a HMOX1-induced tauopathy mechanism [40].

There is growing demand for biomarkers to diagnose AD in its early stages and monitor disease progression. Such biomarkers should ideally be sensitive, specific, and measurable using non-invasive, inexpensive, and easy-to-use techniques. Thus far, the analysis of Aβ_1–42_ as well as total tau and phosphor-tau-181 in CSF is considered the only reliable, sensitive, and specific method for diagnosing AD [41]. However, screening AD patients and monitoring disease progression is difficult due to the invasiveness of CSF collection via lumbar puncture. Thus, many efforts have been made to discover reliable blood-based biomarkers.

Epigenetic alterations have a well-established role in cancer. In particular, hypermethylation of CpG islands in certain genes is one of the most prevalent molecular markers for a variety of human cancers [42]. These epigenetic alterations have broad potential to serve as biomarkers for early diagnosis, prognosis, patient stratification, and prediction of treatment response [43]. Epigenetic biomarkers have several advantages. Patterns of DNA methylation are relatively stable and easy to detect using advanced technologies, such as MSP and high-performance liquid chromatography, and can be measured from a wide variety of specimens, including tissue, plasma, saliva, and urine [42, 44]. Furthermore, alterations in the status of DNA methylation occur during early stages of disease development, allowing for the development of diagnostic biomarkers for early disease detection [42, 44].

We evaluated the DNA methylation status of *HMOX1* at the −374 promoter CpG site as a blood-based biomarker for predicting AD progression. We found that the methylation status of *HMOX1* in the blood can differentiate AD patients from MCI patients and control individuals. Although further large-scale clinical research is warranted, our findings suggest that the methylation status of *HMOX1* at a specific promoter CpG site is related to AD progression.

## Supporting Information

S1 Fig The power of detecting a significant mean difference in methylation in all pairs of AD, MCI and control groups. (A) AD and Control (B) AD and MCI (C) MCI and Control. AD, Alzheimer’s disease; MCI, mild cognitive impairment.
(PDF)

S2 Fig Effects of (A) gender and (B) APOE ε4 allele on DNA methylation level of *HMOX1* gene. M, male; F, female.
(PDF)

S3 Fig The association between age and DNA methylation level of *HMOX1* gene or MMSE score. (PDF)

S4 Fig Analyses of *HMOX1* mRNA expression for multiple brain regions from control and late onset AD patients using public datasets, GSE15222 and GSE5281. EC, entorhinal cortex; HIP, hippocampus; MTG, middle temporal gyrus; PC, posterior cingulate cortex; SFG, superior frontal gyrus; VCX, primary visual cortex; AD, Alzheimer’s disease.
(PDF)

S1 Table Clinical information of blood samples. (PDF)
