# Supplementary figures and images for: Amyloid Beta-Mediated Hypomethylation of Heme Oxygenase 1 Correlates with Cognitive Impairment in Alzheimer’s Disease
Source: PLoS One. 2016 Apr 8;11(4):e0153156. doi: 10.1371/journal.pone.0153156 (PMC4825942; doi:10.1371/journal.pone.0153156)

(A)

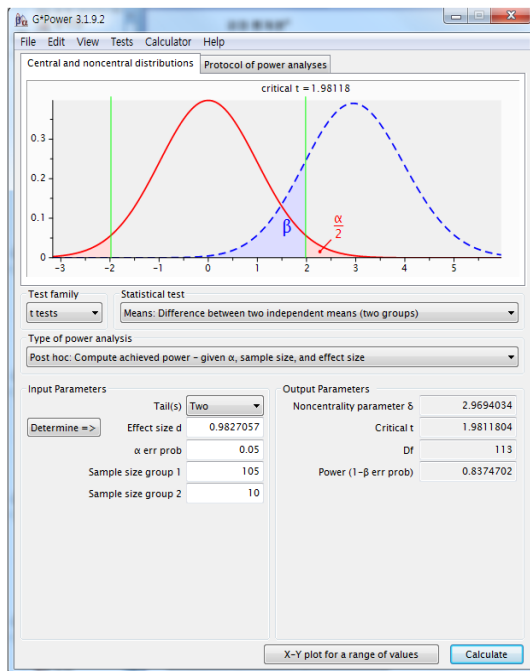

(B)

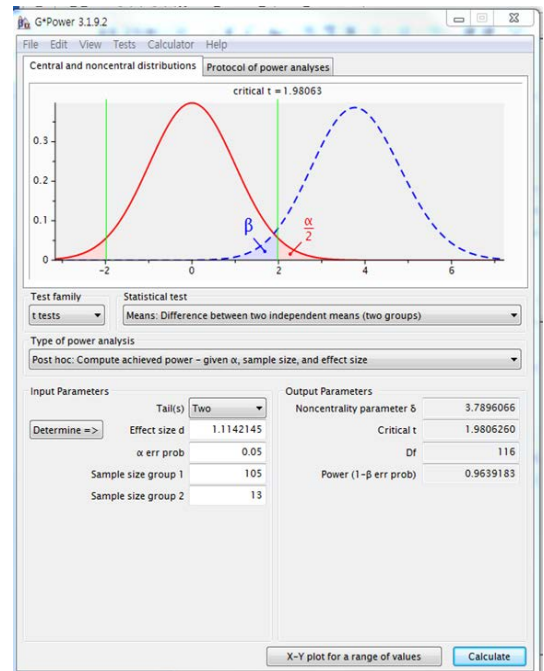

(C)

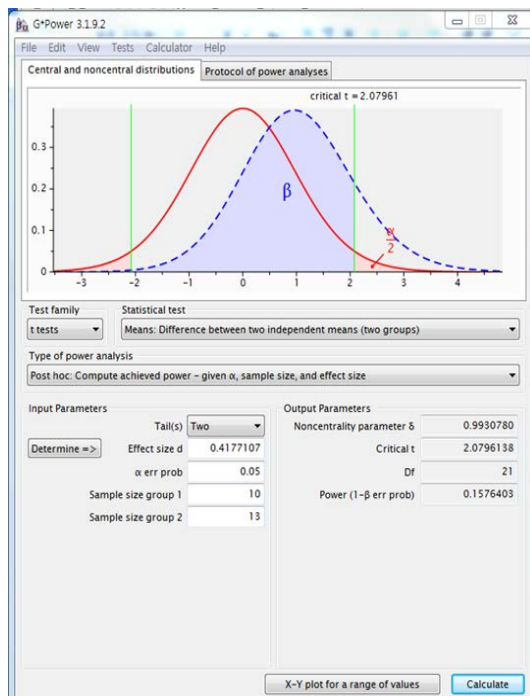

Supplement: S1 Fig — AD, Alzheimer’s disease; MCI, mild cognitive impairment. (PDF) [file pone.0153156.s001.pdf]

**A**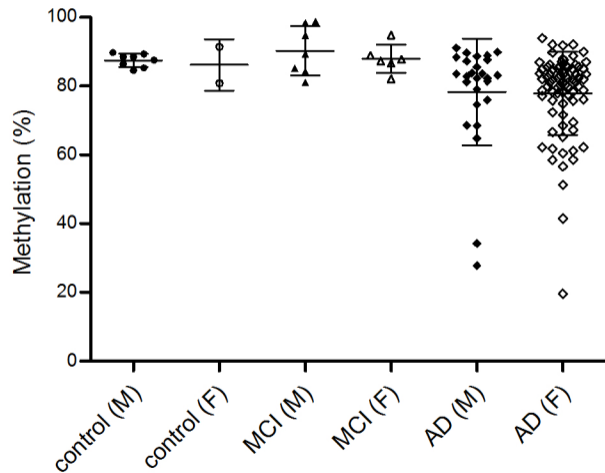**B**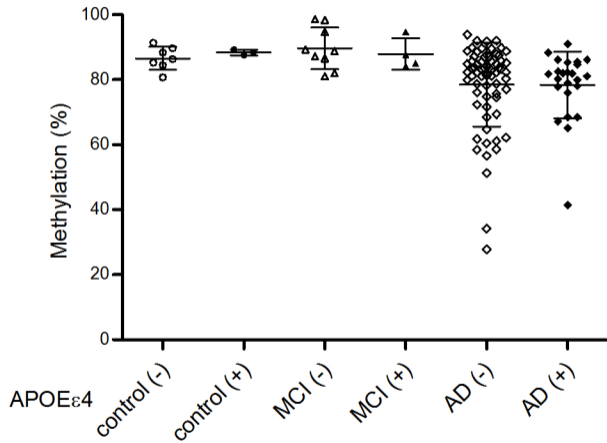

Supplement: S2 Fig — M, male; F, female. (PDF) [file pone.0153156.s002.pdf]

**A**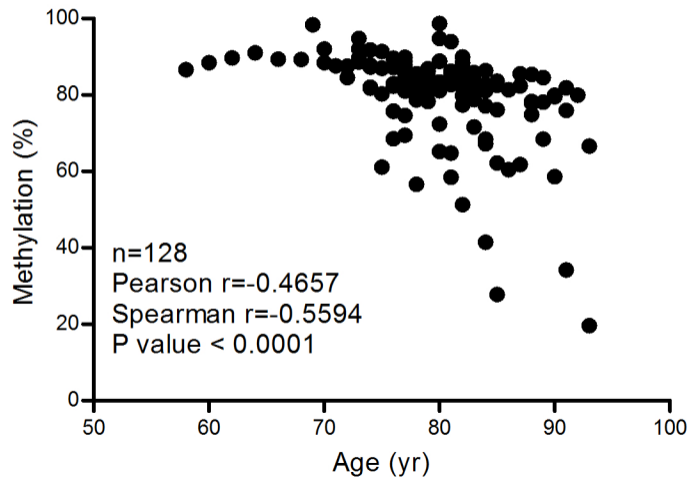**B**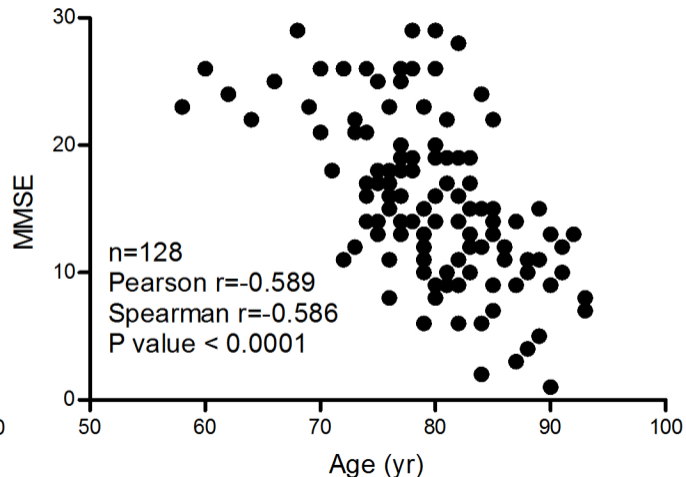

Supplement: S3 Fig — (PDF) [file pone.0153156.s003.pdf]

## GSE15222: brain cortical samples

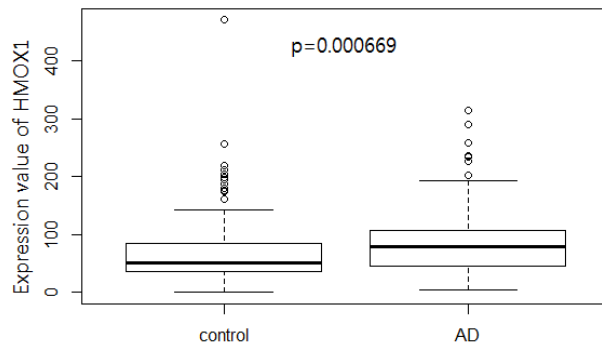

## GSE5281 : 6 brain regions

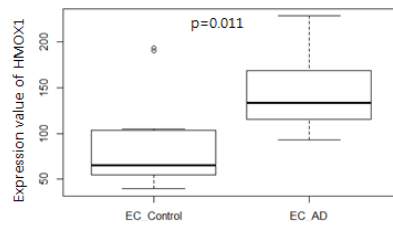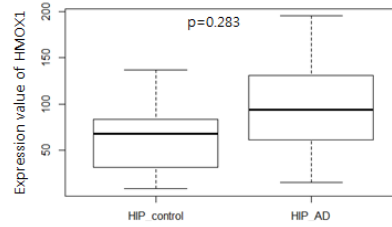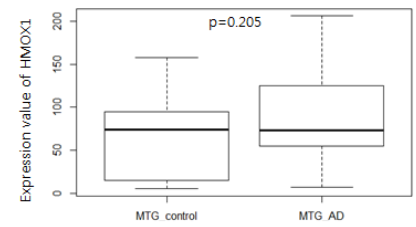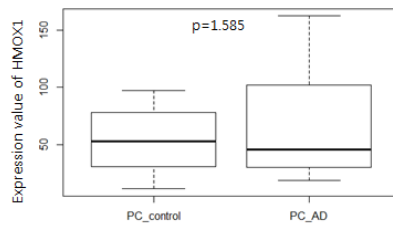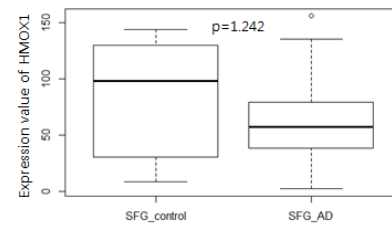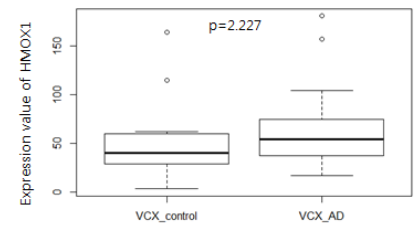

Supplement: S4 Fig — EC, entorhinal cortex; HIP, hippocampus; MTG, middle temporal gyrus; PC, posterior cingulate cortex; SFG, superior frontal gyrus; VCX, primary visual cortex; AD, Alzheimer’s disease. (PDF) [file pone.0153156.s004.pdf]
