# Supplementary material for: Amyloid Beta-Mediated Hypomethylation of Heme Oxygenase 1 Correlates with Cognitive Impairment in Alzheimer’s Disease
Source: PLoS One. 2016 Apr 8;11(4):e0153156. doi: 10.1371/journal.pone.0153156 (PMC4825942; doi:10.1371/journal.pone.0153156)
Supplement: S1 Table — (PDF) [file pone.0153156.s005.pdf]

S1 Table. Clinical information of blood samples

	MMSE	age	gender	ApoE genotype[a]
CON1	29	78	F	E3/E3
CON2	29	68	M	E3/E4
CON3	26	74	M	E3/E4
CON4	26	70	M	E2/E4
CON5	26	72	M	E3/E3
CON6	26	78	M	E3/E3
CON7	26	60	M	E3/E3
CON8	25	75	F	E3/E3
CON9	24	84	M	E3/E3
CON10	24	62	M	E3/E3
MCI1	29	80	M	E3/E3
MCI2	26	80	M	E3/E3
MCI3	25	66	M	E3/E3
MCI4	25	77	M	E3/E4
MCI5	23	69	M	E3/E3
MCI6	23	58	F	E3/E3
MCI7	22	73	F	E3/E4
MCI8	21	74	F	E3/E3
MCI9	21	74	F	E3/E4
MCI10	20	77	F	E3/E3
MCI11	19	78	M	E3/E4
MCI12	18	77	M	E3/E3
MCI13	16	74	F	E3/E3
AD1	28	82	M	E3/E4
AD2	26	77	F	E3/E3
AD3	23	79	M	E3/E3
AD4	23	76	M	E3/E3
AD5	23	79	M	E3/E3
AD6	22	81	F	E2/E3
AD7	22	85	M	E3/E3
AD8	22	64	M	E4/E4
AD9	21	73	F	E3/E3
AD10	21	70	F	E3/E3
AD11	21	73	M	E3/E3
AD12	20	80	M	E3/E3
AD13	20	80	M	E3/E3
AD14	19	83	F	E3/E3
AD15	19	77	F	E3/E3
AD16	19	82	F	E3/E4
AD17	19	81	F	E3/E3
AD18	19	78	F	E3/E4
AD19	19	80	F	E3/E3
AD20	19	81	M	E3/E3
AD21	19	78	F	E3/E3
AD22	18	75	F	E3/E3
AD23	18	78	M	E3/E3
AD24	18	78	F	E3/E4
AD25	18	71	M	E3/E3
AD26	18	77	M	E3/E3
AD27	18	76	F	E3/E4
AD28	17	83	F	E3/E3
AD29	17	83	F	E3/E3
AD30	17	75	F	E3/E3
AD31	17	74	F	E3/E3
AD32	17	76	M	E3/E4
AD33	17	81	F	E4/E4
AD34	16	76	F	E3/E3
AD35	16	82	F	E3/E3
AD36	16	77	M	E3/E3
AD37	16	80	F	E3/E4
AD38	15	76	M	E3/E3
AD39	15	83	M	E3/E3
AD40	15	89	F	E3/E3
AD41	15	84	F	E3/E3
AD42	15	84	F	E3/E3
AD43	15	85	F	E3/E4
AD44	15	79	F	E3/E3
AD45	14	74	F	E3/E4
AD46	14	87	M	E3/E3
AD47	14	82	F	E3/E3
AD48	14	85	F	E3/E3
AD49	14	75	F	E4/E4
AD50	14	78	F	E3/E3
AD51	14	77	F	E3/E3
AD52	14	80	F	E3/E3
AD53	13	79	M	E3/E3
AD54	13	79	F	E3/E3
AD55	13	79	F	E3/E3
AD56	13	90	F	E3/E3
AD57	13	92	F	E3/E4
AD58	13	77	F	E3/E3
AD59	13	75	F	E3/E3
AD60	13	83	M	E3/E4
AD61	13	83	F	E3/E3
AD62	13	85	F	E3/E3
AD63	12	91	M	E3/E4
AD64	12	86	M	E3/E3
AD65	12	84	F	E3/E4
AD66	12	79	F	E3/E3
AD67	12	83	F	E3/E3
AD68	12	73	F	E3/E3
AD69	12	84	F	E3/E4
AD70	12	91	M	E3/E3
AD71	11	89	F	E3/E4
AD72	11	79	F	E3/E4
AD73	11	82	F	E3/E3
AD74	11	79	F	E4/E4
AD75	11	88	F	E3/E4
AD76	11	76	F	E3/E3
AD77	11	72	F	E3/E3
AD78	11	86	F	E3/E3
AD79	10	83	M	ND
AD80	10	81	F	ND
AD81	10	91	F	ND
AD82	10	88	F	E3/E3
AD83	10	79	F	ND
AD84	9	85	F	E3/E3
AD85	9	87	F	E3/E3
AD86	9	82	F	E3/E3
AD87	9	81	F	E3/E3
AD88	9	80	F	E3/E3
AD89	9	90	F	ND
AD90	8	80	F	E3/E4
AD91	8	93	F	ND
AD92	8	76	F	ND
AD93	7	93	F	ND
AD94	7	85	F	ND
AD95	6	82	F	ND
AD96	6	84	M	ND
AD97	6	79	F	ND
AD98	6	82	F	ND
AD99	6	82	F	ND
AD100	5	89	F	E3/E3
AD101	4	88	F	E3/E3
AD102	4	88	F	E3/E4
AD103	3	87	F	E3/E3
AD104	2	84	M	E3/E4
AD105	1	90	F	E3/E3

M, Male; F, female; AD, Alzheimer's disease; MCI, mild cognitive impairment; MMSE, mini-mental state examination; ND, not deteected

[a]APOE information from 14 patients of AD is missing
